# Artificial intelligence in thyroid ultrasound: clinical applications and perspectives

**DOI:** 10.3389/fendo.2026.1877780

**Published:** 2026-08-11

**Authors:** Ye Guo, Tong Zhao, Lili Zhang, Yansong Liu, Kai Liu, Beibei Han, Yiming Zhao, Jiangbo Shao, Lirong Zhao

**Affiliations:** 1 Cancer Center, The First Hospital of Jilin University, Changchun, Jilin, China; 2 Imaging Center, The Third Affiliated Hospital of Changchun University of Chinese Medicine, Changchun, Jilin, China; 3 Ultrasound Diagnostic Center, The First Hospital of Jilin University, Changchun, Jilin, China; 4 Department of Hand and Foot Surgery, Orthopedics Center, The First Hospital of Jilin University, Changchun, Jilin, China

**Keywords:** artificial intelligence, clinical translation, risk stratification, thyroid nodule, thyroid ultrasound

## Abstract

Thyroid nodules are highly prevalent, with increasing detection rates driven by advanced imaging and expanded screening. Ultrasound serves as the first-line tool for screening, diagnosis and follow-up, owing to its non-invasiveness, real-time capability, cost-effectiveness and absence of ionizing radiation. However, conventional ultrasound diagnosis is highly operator-dependent, resulting in substantial inter-observer variability and diagnostic errors, particularly for subtle or indeterminate lesions. Artificial intelligence (AI), particularly deep learning and radiomics, has emerged as a promising approach to address these limitations by enabling automated feature extraction, quantitative analysis and standardized interpretation, which has the potential to improve diagnostic efficiency and risk stratification. This review summarizes AI applications in thyroid ultrasound, including image preprocessing, nodule segmentation, quantitative feature analysis, benign-malignant differentiation, TIRADS optimization and automated reporting. We highlight AI’s potential in enhancing diagnostic consistency and accuracy, while critically assessing the methodological quality, bias risks and external validation of existing studies. Most AI tools are still in early translational phases, lacking large-scale validation in real clinical settings and standardized reporting protocols. We further discuss key challenges, including data bias, limited generalizability due to small or single-center datasets, poor interpretability and significant translational barriers. Future directions involving multi-modal fusion, explainable AI, real-time clinical systems and rigorous, multi-center standardized validation are proposed to facilitate clinical translation and improve patient care.

## 1 Introduction

### 1.1 Clinical status and challenges of thyroid ultrasound

Thyroid disorders, including nodular disease, autoimmune thyroiditis, and thyroid cancer, are prevalent worldwide, and thyroid nodules rank among the most common endocrine diseases in clinical practice (1). The prevalence of thyroid nodules in the general population is remarkably high, with ultrasound studies detecting nodules in a substantial proportion of individuals—far exceeding the detection rate of palpable nodules during physical examination (2). While the vast majority of thyroid nodules are benign, the potential for malignancy necessitates accurate and timely differentiation to avoid over-treatment or under-treatment in routine clinical practice. Thyroid cancer incidence has also shown a steady upward trend in recent decades, driven in part by improved detection of small, indolent tumors through widespread ultrasound screening, as well as potential changes in environmental and genetic risk factors (3).

Ultrasound is the primary modality for thyroid nodule evaluation, featuring non-invasiveness, wide accessibility, real-time imaging, low cost and good repeatability (4). It is widely deployed in real-world clinical settings and applied across the whole clinical workflow, including population screening, lesion assessment, fine-needle aspiration (FNA) guidance and long-term follow-up. Different from CT and MRI, ultrasound delivers detailed anatomical and functional information without ionizing radiation. It is suitable for repeated examinations and screening for children, adolescents and pregnant women. It also assists in evaluating autoimmune thyroiditis and identifying nodules hidden by diffuse parenchymal changes (5).

Despite these significant merits, conventional ultrasound diagnosis is confronted with substantial challenges that compromise its reliability and reproducibility (6). The primary limitation is the inherent subjectivity of image interpretation, which is also a major source of clinical assessment bias. The assessment of key ultrasound features is highly dependent on the individual experience, training, and visual perception of the clinician (7). Studies have consistently demonstrated moderate to fair inter-observer agreement for these critical malignant risk features among experienced and senior radiologists, leading to inconsistent diagnostic conclusions and clinical decisions across different institutions and providers. This variability is particularly pronounced for subtle or borderline features, which are often critical for identifying early-stage thyroid cancer or distinguishing benign lesions from indolent malignancies. For novice physicians, the learning curve associated with mastering ultrasound image interpretation is notably steep, often requiring years of clinical practice to achieve diagnostic accuracy comparable to that of senior colleagues. This discrepancy in expertise contributes to healthcare disparities, with patients in resource-limited settings or those cared for by less experienced clinicians facing higher risks of misdiagnosis (8).

A second major challenge is the lack of uniform standards governing the global application of diverse Thyroid Imaging Reporting and Data System (TIRADS), including ACR-TIRADS, EU-TIRADS, K-TIRADS and C-TIRADS (9). Multiple TIRADS frameworks have been developed globally, each with distinct criteria for risk stratification and management recommendations, resulting in inconsistent reporting norms across clinical studies. The inconsistent adoption of these systems across institutions leads to fragmented clinical decision-making, with some nodules being overclassified as high-risk and others under classified. This lack of standardization confuses clinicians and brings uncertainty to patients who receive conflicting recommendations from different medical providers.

Workflow inefficiencies further restrict the clinical utility of thyroid ultrasound in high-volume medical centers (10). Manual measurement of nodule dimensions, comprehensive documentation of multiple ultrasonic features, and the compilation of diagnostic reports consume abundant clinical time. The heavy workload becomes a prominent bottleneck in tertiary hospitals and community screening programs where ultrasound specialists are insufficient. Meanwhile, centralized storage and longitudinal comparative analysis of massive ultrasound image data create logistical difficulties for medical facilities with outdated information infrastructure (11).

Finally, diagnostic uncertainty surrounding grey zone nodules with indeterminate FNA cytology remains a persistent clinical difficulty (12). A considerable proportion of FNA specimens generate equivocal results, which force patients to receive additional molecular testing, repeated biopsy or surgical resection (13). These follow-up procedures raise overall medical expenditure and induce persistent patient anxiety (14). Conventional ultrasound lacks sufficient discriminatory power for such indeterminate lesions, creating an unmet clinical demand for auxiliary diagnostic tools (15).

### 1.2 Rationale for AI intervention and technical basis

The rapid development of artificial intelligence has transformed analytical paradigms across medical imaging disciplines (15). AI can simulate human learning and pattern recognition capacity, and efficiently extract complex, subtle imaging patterns that human observers may overlook. Such strengths can effectively offset the core drawbacks of conventional thyroid ultrasound interpretation and mitigate clinical assessment bias. Machine learning and deep learning-based analytical tools have achieved promising outcomes in lesion detection, tissue classification and prognostic analysis within radiology, pathology and cardiology (16). For thyroid ultrasound scenarios, AI systems can lower inter-observer deviation and improve diagnostic stability for clinicians at all experience tiers (17).

Two core technical frameworks underpin AI research on thyroid ultrasound: deep learning and radiomics (18). Deep learning relies on multi-layer neural network architectures to realize automatic imaging feature extraction without manual feature engineering conducted by clinical experts (19). Convolutional Neural Networks (CNNs) are the most widely adopted deep learning backbone for thyroid ultrasound analysis, undertaking core tasks including nodule detection and benign-malignant classification (20).

Radiomics pipelines extract hundreds to thousands of quantitative morphological, textural and grayscale metrics from segmented nodule regions (21). Different from end-to-end deep learning models, radiomics outputs traceable, interpretable imaging signatures that can be correlated with pathological outcomes, which helps build clinician trust during clinical translation (22). Existing retrospective single-center research reports AUC values between 0.85 and 0.94 for AI nodule risk discrimination, yet model performance regularly drops during independent multi-center external validation, which reflects widespread selection bias and limited cross-population generalizability (22). Existing research confirms that AI can boost the identification rate of microcalcifications and optimize triage efficiency for indeterminate nodules, yet nearly all available evidence originates from single-center retrospective cohorts without prospective real-world clinical verification (23). The construction of multi-center annotated imaging datasets and popularization of high-performance GPU computing have accelerated iterative optimization of AI prototypes, while heterogeneous scanning equipment and variable patient populations still restrict uniform cross-hospital deployment (24).

### 1.3 Objectives and scope of this narrative review

This narrative review consolidates landmark original clinical research and professional consensus statements focusing on artificial intelligence for thyroid ultrasound, without quantitative meta-analysis or pooled evidence synthesis. Formal systematic review workflows, exhaustive database retrieval protocols and quantitative bias evaluation tools such as QUADAS-2 are not adopted in this manuscript. We selectively incorporate peer-reviewed clinical studies with pathological validation, while excluding purely algorithmic engineering papers without corresponding thyroid ultrasound clinical data. Primary original research and secondary meta-analytic literature are discussed separately in subsequent sections to avoid mixing different levels of clinical evidence.

The core discussion domains of this review cover image preprocessing and artifact suppression, thyroid nodule segmentation and localization, automated quantitative extraction of ultrasonic markers, AI-assisted benign-malignant differentiation, optimized TIRADS risk grading, and computer-aided structured diagnostic reporting. We elaborate the core operating logic of deep learning and radiomic architectures, alongside dataset construction standards and multi-stage model training & validation workflows. This review delivers balanced critical discussion of pervasive technical and translational obstacles, including dataset bias, insufficient coverage of rare pathological subtypes, poor model interpretability, workflow integration barriers, regulatory limitations and unresolved ethical and legal risks. This synthesis targets a multidisciplinary readership consisting of endocrinologists, diagnostic sonographers, translational imaging researchers and medical device policymakers. Throughout the full text, we consistently clarify that AI only serves as auxiliary clinical reference rather than a replacement for independent clinician judgment.

Literature supporting this narrative synthesis was retrieved via targeted searches across PubMed, Embase, Web of Science and Scopus for peer-reviewed English manuscripts published up to March 30, 2026. Selection followed a selective rather than exhaustive framework: we prioritized clinical original studies, meta-analyses and cross-institutional consensus statements with pathological validation for thyroid ultrasound AI pipelines. Manuscripts confined to abstract-only records, non-English full texts, and pure algorithmic simulations without human imaging cohorts were omitted from qualitative discussion. No standardized systematic review protocols or quantitative bias assessment instruments were applied throughout literature curation. **Table 1** only enumerates landmark representative studies for intuitive cross-comparison, rather than a full inventory of all retrieved publications. All evidence integration relies on thematic narrative appraisal, without pooled quantitative meta-analytic synthesis.

**Table 1:** Summary of representative artificial intelligence models for thyroid ultrasound auxiliary diagnosis.

References	Model type	Core task	Main performance	Validation type	Overall study rigor	Main limitations
(33)	U-Net	Nodule segmentation	DSC > 0.8	Multi-center external validation	Moderate	Poor performance on low-quality/blurred images
(34, 35)	ResNet	Nodule classification	AUC = 0.85–0.94	Single-center internal validation	Low	Limited generalizability across diverse clinical settings
(50)	Radiomics	Benign-malignant differentiation	AUC > 0.87	Single-center internal validation	Low	Performance highly dependent on accurate ROI segmentation
(72)	CDAE, GAN	Image denoising & contrast enhancement	Improved SNR and feature visibility	Single-center internal validation	Low	Inconsistent experimental settings lead to poor repeatability
(74)	Deep learning	Artifact detection & removal	High accuracy for common artifacts	Single-center internal validation	Low	Lack of unified assessment standards for artifacts
(79, 80)	YOLO, Faster R-CNN	Nodule detection	Sensitivity > 0.88	Single-center internal validation	Moderate	Insufficient validation in primary care settings
(82, 83)	CNN	Automatic feature extraction	High inter-rater consistency	Single-center internal validation	Low	Non-unified feature extraction standards
(94)	Hybrid model (DL + Radiomics)	TIRADS classification	AUC > 0.91	Multi-center external validation	Moderate	Difficulties in real-world deployment
(100)	End-to-end AI system	Automated structured reporting	High standardization rate	Single-center internal validation	Low	No unified templates across institutions
(123)	XAI (Attention + NLP)	Model interpretability optimization	Improved clinical readability	Single-center internal validation	Low	Lack of unified evaluation metrics for interpretability

This table only summarizes representative landmark studies for qualitative narrative comparison, without full inventory of all retrieved literature. This manuscript does not apply quantitative bias evaluation tools or formal systematic review frameworks; study heterogeneity is discussed descriptively in subsequent text.

## 2 Core technical principles of AI in thyroid ultrasound

Comprehensive understanding of fundamental AI architectures, dataset construction criteria and model iteration pipelines is essential to analyze translational gaps between computational prototypes and routine thyroid ultrasound clinical practice (25). This section introduces two mainstream technical paradigms, standardized data quality control pipelines, and multi-stage training-validation frameworks widely applied to thyroid nodule analytical models.

### 2.1 Mainstream technical frameworks

**Figure 1:**
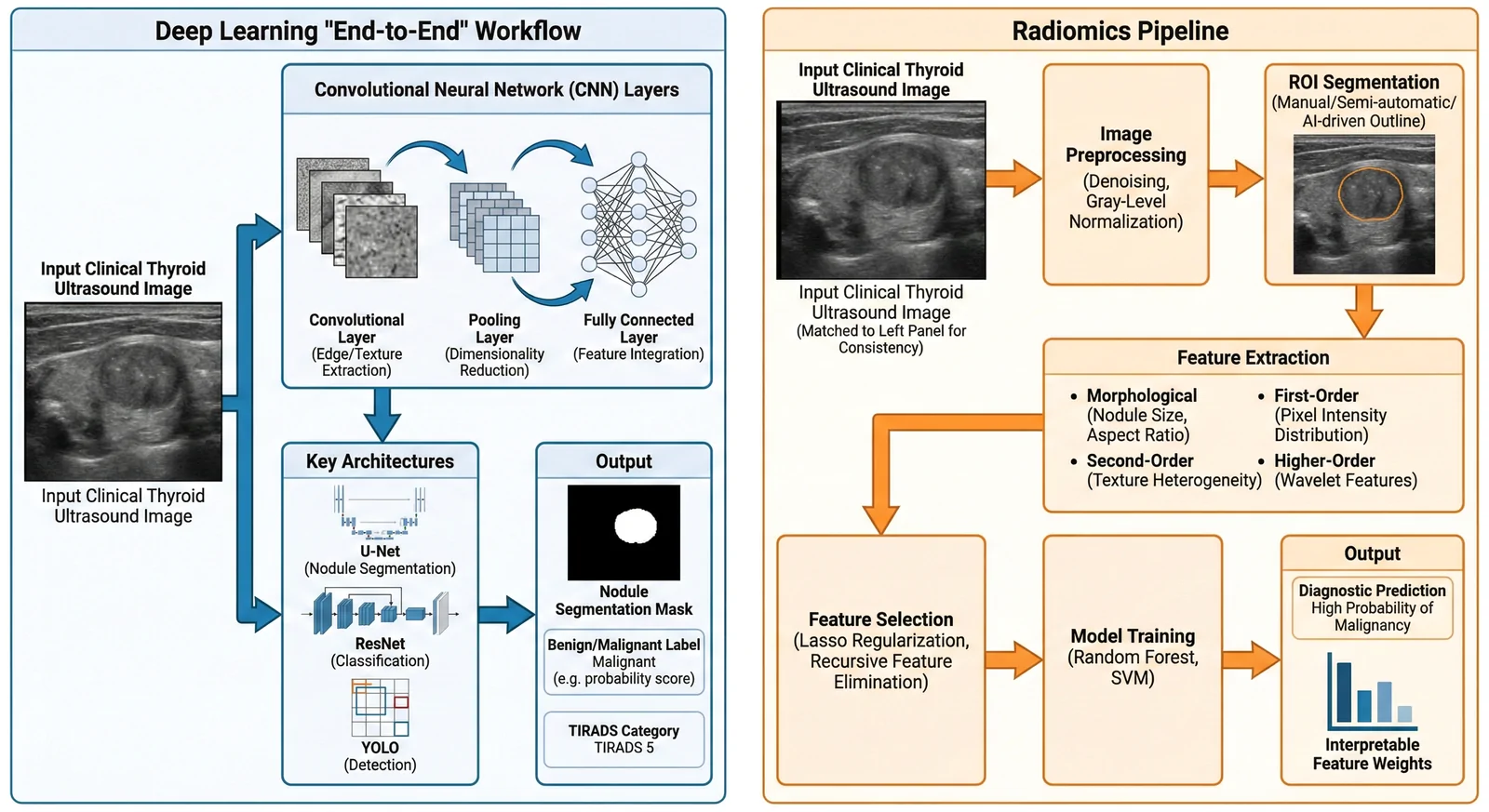
Schematic comparison of deep learning end-to-end pipeline and multi-step radiomics workflow for thyroid ultrasound analysis.

The application of AI in thyroid ultrasound is anchored by two core technical frameworks: deep learning and radiomics (2, 26). Each paradigm offers distinct advantages and has been extensively applied to address specific challenges in thyroid ultrasound analysis (**Figure 1**).

#### 2.1.1 Deep learning

Deep learning is a branch of machine learning built by stacked neural layers, which can autonomously extract multi-layer spatial imaging features without manual feature engineering by clinical specialists (27). After training on annotated ultrasound cohorts, convolutional neural networks (CNNs) become the most widely adopted backbone for thyroid imaging analysis, capturing multi-scale morphological and textural features embedded in two-dimensional grayscale ultrasound images (28, 29). CNNs support three core clinical tasks: whole-image classification, multi-lesion object detection, and pixel-wise nodule segmentation (30). Accurate pixel-wise segmentation supports precise nodule volume measurement and longitudinal lesion comparison, which acts as the precondition of subsequent quantitative feature extraction (31). A variety of CNN variants have been optimized to solve unique thyroid imaging challenges, with differentiated structural advantages for different clinical tasks (32).

U-Net and its encoder-decoder derivative models retain fine lesion boundary information through skip connections, realizing high-precision segmentation of small nodules with irregular contours (33). ResNet introduces residual shortcut structures to solve gradient vanishing problems, enabling training of deep networks optimized for nodule risk stratification and feature discrimination (34, 35). DenseNet strengthens inter-layer feature reuse to stabilize model performance under limited annotated ultrasound datasets (36). Real-time detection frameworks including YOLO and Faster R-CNN realize rapid multi-nodule localization, adapting to high-throughput population screening workflows (37).

Transfer learning mitigates the core bottleneck of insufficient annotated clinical thyroid ultrasound data (38). Pre-trained weights obtained from general public image datasets are fine-tuned on thyroid imaging cohorts, reducing computational training costs and improving baseline generalization performance (39). However, most transfer learning models are only validated in single institutional internal datasets, lacking cross-center performance verification. Weakly supervised and semi-supervised learning further reduce manual annotation burden by utilizing partially labeled or unannotated ultrasound scans, yet unstable prediction consistency remains when processing heterogeneous real-world clinical images (40).

#### 2.1.2 Radiomics

Radiomics extracts hundreds of interpretable high-dimensional quantitative imaging signatures from delineated nodule regions, capturing microstructural heterogeneity invisible to human visual observation (41). Different from end-to-end deep learning pipelines, radiomics follows a fixed multi-stage workflow: image normalization, ROI segmentation, feature filtration and traditional machine learning modeling (42).

Preprocessing unifies grayscale distribution, suppresses equipment-specific noise and eliminates intensity deviation caused by inconsistent scanning parameters. Nevertheless, inconsistent normalization and denoising protocols adopted across independent research groups lead to prominent inter-study heterogeneity, which hinders direct horizontal comparison of model performance (43) ROI segmentation is a prerequisite for reliable signature extraction, which can be completed via manual drawing, semi-automatic segmentation or U-Net-based automatic delineation. Manual segmentation inevitably introduces inter-operator deviation and massive time costs when processing large imaging cohorts (44, 45).

Four categories of quantitative descriptors are commonly extracted from segmented nodule regions: morphological metrics, first-order pixel intensity statistics, second-order texture descriptors, and high-order wavelet or fractal features (46, 47). High-dimensional feature sets carry inherent overfitting risks; dimensionality reduction strategies such as Lasso regularization and recursive feature elimination are thus adopted to retain only predictive imaging biomarkers (48). Filtered feature vectors are input into classical classifiers including support vector machines and random forests to construct pathological risk prediction models (49).

Radiomic pipelines achieve competitive auxiliary discrimination performance for cytologically indeterminate thyroid nodules, but their diagnostic robustness heavily relies on accurate ROI delineation. Excess feature dimensionality and single-center training cohorts frequently trigger overfitting, restricting stable generalization performance in diversified real clinical environments (50).

### 2.2 Data requirements and quality control

**Figure 2:**
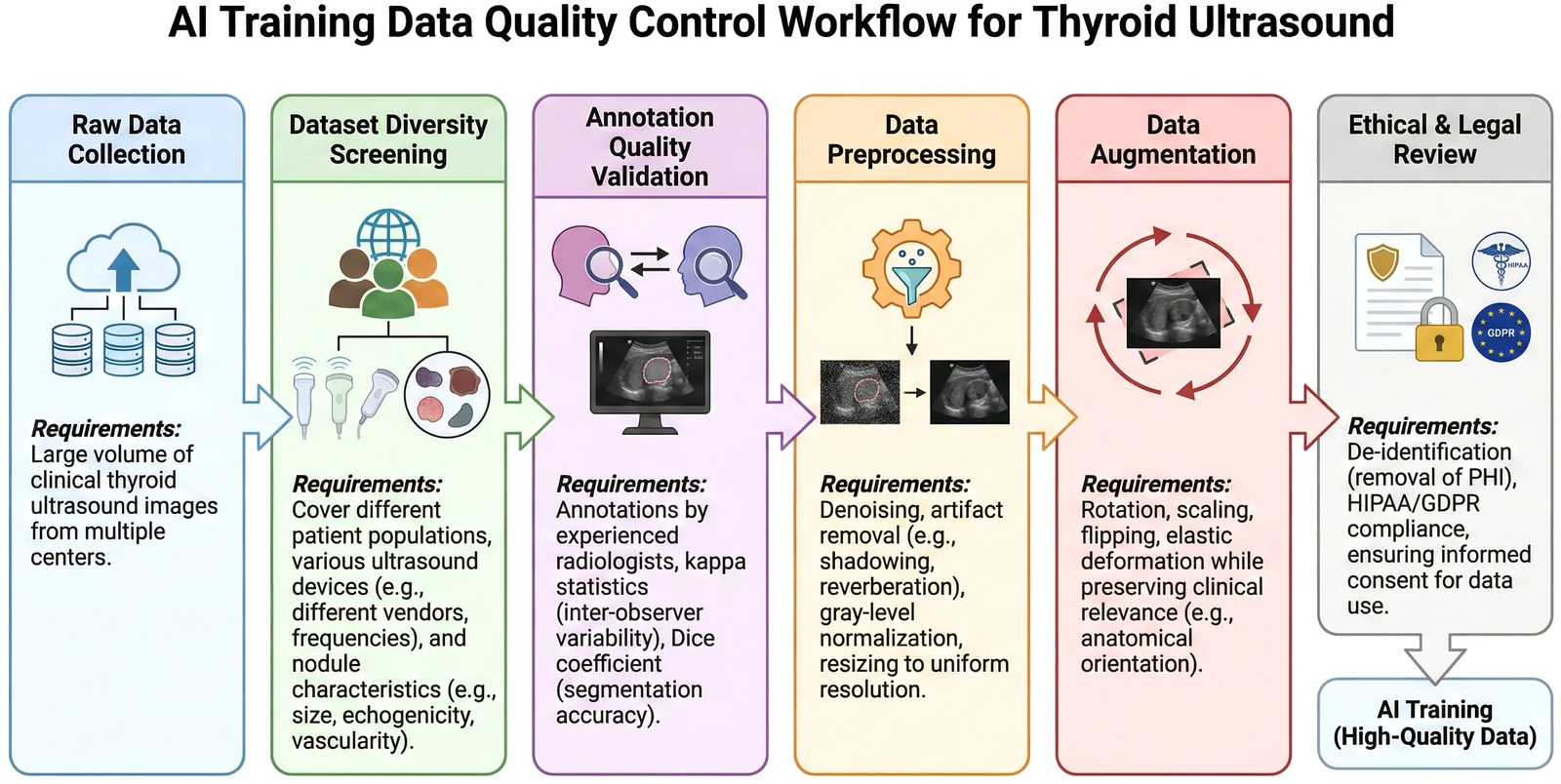
Full workflow of quality control for thyroid ultrasound AI training datasets.

The performance of AI models in thyroid ultrasound is highly dependent on the quality, quantity, and diversity of training data (51). To develop robust, generalizable models that perform well in real-world clinical settings, several key considerations must be addressed (**Figure 2**).

#### 2.2.1 Dataset size and diversity

Deep neural network analytical models require large-scale fully annotated clinical imaging cohorts to avoid simply memorizing dataset-specific noise artifacts and local image characteristics rather than learning universal pathological imaging patterns applicable to general thyroid nodule populations (52). Single-institution self-built datasets inevitably generate inherent selection bias originating from limited ultrasound equipment types, narrow local patient demographic composition and region-specific thyroid disease prevalence characteristics. Multi-center collaborative integrated datasets effectively mitigate dataset shift effects by incorporating variable ultrasound scanning hardware and highly heterogeneous multi-regional patient populations with distinct nodule pathological subtypes (53). Comprehensive high-quality cohort construction needs to fully cover the complete spectrum of clinical nodule characteristics, including nodule size gradient, internal echogenicity classification, calcification morphological patterns and all distinct rare and common pathological subtypes; standardized shared public multi-center annotated thyroid ultrasound datasets remain extremely scarce across global academic research consortia (54).

#### 2.2.2 Annotation quality

All supervised deep learning and radiomics model training processes completely depend on standardized ground truth image labels manually annotated by experienced professional thyroid radiologists with abundant clinical diagnostic experience (55). Multiple inter-annotator consistency quantitative metrics including Dice similarity coefficient and Kappa consistency value are adopted to quantify labeling concordance between different radiologists; multi-expert consensus collaborative labeling strategies are widely adopted to resolve discrepant judgements on lesion boundary ranges and ultrasonic feature definition standards (56). All valid annotated image labels must be cross-referenced with postoperative surgical pathological results or fine-needle aspiration cytological reports as uniform gold diagnostic standards to eliminate systematic labeling bias. However, numerous published research works omit complete explicit reporting of inter-rater consistency quantitative evaluation indicators in their manuscripts.

#### 2.2.3 Data preprocessing and augmentation

Raw thyroid clinical ultrasound images collected from routine clinical work are frequently contaminated by multiple types of interference artifacts including motion blur, acoustic calcification shadow and reverberation noise, all of which will interfere with stable feature learning of neural network models (57). Standardized unified denoising, targeted artifact removal and uniform spatial resampling operations normalize the overall input image gray-scale distribution for model training, while geometric data augmentation operations including rotation, flipping and scaling artificially expand effective cohort sample volume without destroying anatomically valid clinical tissue feature information (58). Non-uniform augmentation operation schemes adopted across different published research studies create inconsistent experimental baseline conditions, which blocks reliable cross-study horizontal performance comparison between different AI models.

#### 2.2.4 Ethical and legal considerations in data use

All patient clinical imaging data utilized for AI model training and research development must fully comply with global universal patient data protection regulatory frameworks such as HIPAA and GDPR, requiring complete de-identification processing of all protected personal health information embedded within ultrasound images (59). Formal written informed consent from participating patients is generally mandatory for centralized aggregation and secondary utilization of clinical imaging data for research purposes, while multiple published academic articles lack complete standardized disclosure of ethical approval documents and detailed patient participant consent records (60). Cross-hospital multi-center data collaborative research demands formal standardized data sharing agreements to clarify patient privacy constraint rules and intellectual property ownership boundaries of imaging datasets.

### 2.3 Model training, validation, and evaluation

**Figure 3:**
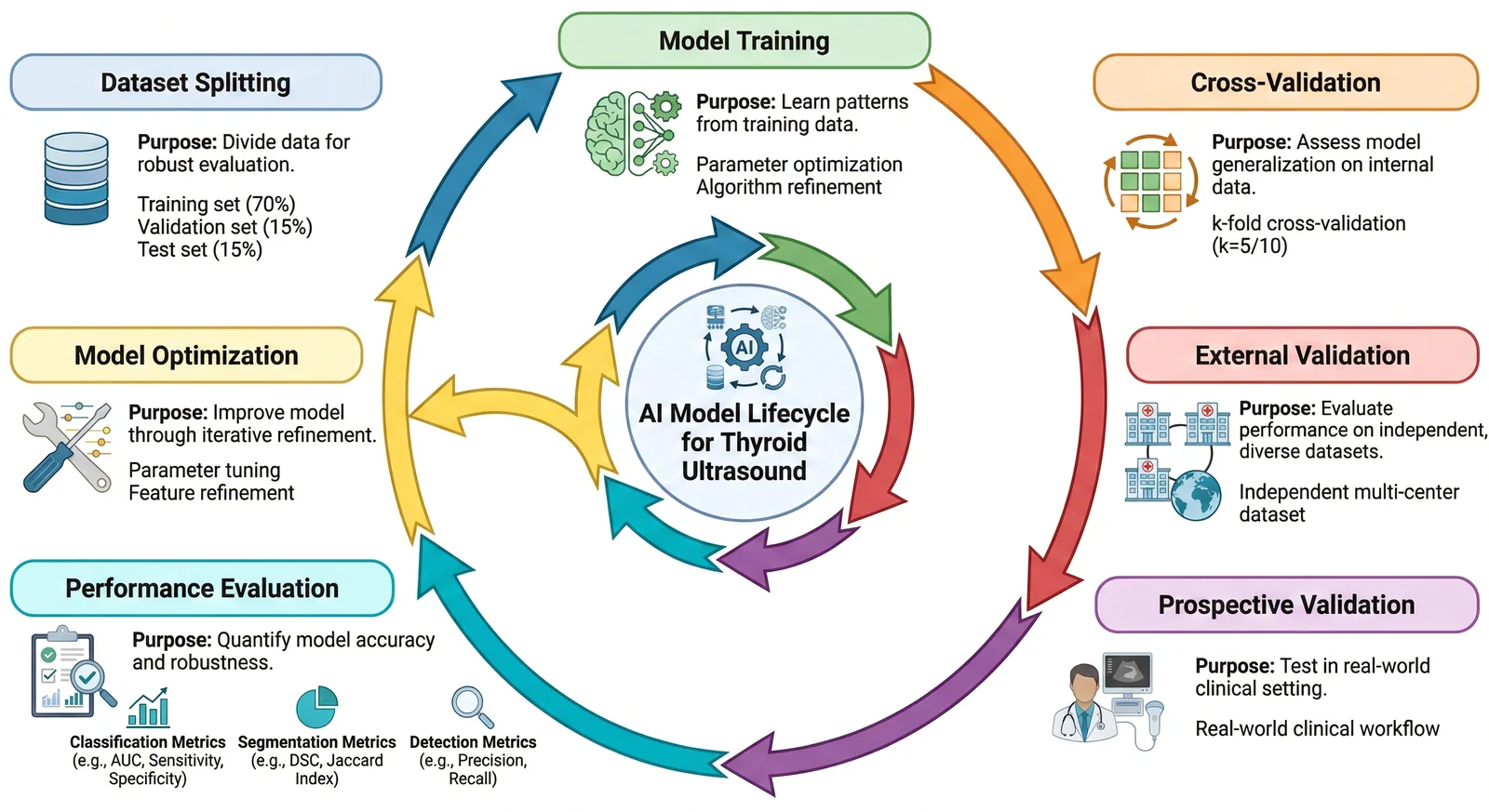
Closed-loop lifecycle of thyroid ultrasound AI model development and verification.

The development of a robust AI model for thyroid ultrasound involves a rigorous process of training, validation, and evaluation—following standards established by medical AI guidelines and regulatory bodies (61). This process ensures that models are accurate, reliable, and generalizable to real-world clinical settings (**Figure 3**).

#### 2.3.1 Training and validation strategies

Annotated complete thyroid ultrasound datasets are rationally partitioned into three mutually exclusive subsets: model training subsets, internal validation subsets and fully unseen independent test subsets, to strictly separate iterative model parameter tuning processes from final objective comprehensive performance assessment (62). K-fold cross-validation analysis provides stable internal performance estimation results under limited total clinical sample sizes with insufficient cohort scale (63). Independent multi-center external validation serves as the core gold standard criterion for evaluating cross-population generalization capacity, testing model diagnostic stability on completely unseen patient clinical cohorts and scanning devices that never participate in model training and parameter optimization (64). Prospective real-world clinical validation represents the most rigorous verification form for AI auxiliary diagnostic models, which tests model practical efficacy within routine daily diagnostic workflows of ultrasound departments. Nevertheless, such prospective validation trials remain extremely resource-intensive and are severely underrepresented in current published thyroid ultrasound AI research (65).

#### 2.3.2 Evaluation metrics

Binary benign-malignant classification models are comprehensively benchmarked via multiple unified diagnostic indicators including AUC, sensitivity, specificity, positive predictive value and negative predictive value (66). Nodule segmentation tasks adopt DSC, Jaccard index and Hausdorff distance to quantitatively measure spatial overlap degree between AI automatic segmentation masks and expert manual ground truth delineations; a DSC value above 0.8 is widely recognized as the threshold of clinically acceptable segmentation precision for thyroid nodules (67). Object detection pipelines rely on precision, recall and F1-score to quantify overall lesion capture efficiency across all ultrasound frames (68). Many existing published research studies only report partial fragmented evaluation indicators rather than complete comprehensive diagnostic metrics, which makes it difficult to draw unified, comparable conclusions when interpreting results from independent single-center trials.

#### 2.3.3 Benchmarking and comparative studies

Multiple controlled head-to-head comparative clinical cohort trials confirm that fully trained and validated AI thyroid ultrasound systems achieve diagnostic accuracy comparable to mid-level or experienced senior radiologists under single-center retrospective research cohort conditions, especially in automatic identification of tiny microcalcifications and standardized risk grading of borderline ambiguous lesions (69). Nevertheless, such relative performance superiority observed in single-center controlled experiments cannot be sustainably maintained under mixed multi-device heterogeneous clinical environments across multiple hospitals. It is clinically critical to repeatedly emphasize that all AI analytical frameworks merely function as auxiliary clinical reference tools rather than full replacements for independent comprehensive clinical judgment of licensed professional clinicians. Most existing comparative diagnostic performance analyses are limited to single tertiary hospital retrospective imaging cohorts without systematic real-world prospective clinical verification.

## 3 Current clinical applications of AI in thyroid ultrasound

AI has been progressively integrated into multiple key segments of routine thyroid ultrasound workflows, covering image optimization, automated lesion detection, quantitative feature analysis, risk stratification and standardized clinical reporting (70). A large body of clinical research has verified the auxiliary diagnostic value of AI in thyroid nodule evaluation. This section narratively summarizes representative published findings and objectively elaborates the practical application scenarios, existing advantages and unresolved clinical limitations of current AI-assisted thyroid ultrasound techniques, without exhaustive literature screening or quantitative pooled analysis.

### 3.1 Image preprocessing and artifact reduction

In routine clinical scanning, thyroid ultrasound images are frequently degraded by motion artifacts, acoustic shadowing, reverberation noise and inconsistent imaging parameters caused by different equipment and scanning habits (71). These interfering factors may obscure subtle malignant features such as tiny microcalcifications and ill-defined margins, thereby affecting clinicians’ comprehensive judgment of nodule risk. To resolve such imaging quality defects, multiple deep learning-based preprocessing methods have been developed to achieve image denoising, contrast enhancement and unified image standardization.

#### 3.1.1 Denoising and contrast enhancement

Convolutional denoising autoencoders and generative adversarial networks are widely adopted for ultrasound image denoising and optimization (72). These models suppress random noise and background interference while retaining diagnostically critical microstructural features, avoiding over-smoothing of lesion edges. Targeted contrast adjustment further optimizes the grayscale distribution of ultrasound images and improves the visual differentiation between nodules and surrounding normal thyroid parenchyma. Such optimization is particularly helpful for identifying hypoechoic lesions and subtle structural abnormalities in heterogeneous parenchyma associated with autoimmune thyroid diseases (73). Nevertheless, most relevant studies are based on single-center retrospective datasets with inconsistent preprocessing pipelines, leading to variable generalization effects in real-world scenarios.

#### 3.1.2 Artifact detection and removal

Common ultrasound artifacts, including reverberation artifacts and posterior acoustic shadows, may simulate malignant sonographic features and increase false-positive diagnostic rates (74). Specialized AI models can automatically identify artifact regions and perform targeted correction without altering valid tissue information in non-artifact areas. This optimization effectively reduces diagnostic misjudgment caused by imaging interference and improves the overall consistency of subsequent feature analysis and risk assessment (75). At present, unified quantitative evaluation standards for artifact correction performance remain absent in this field, and relevant technical verification mainly relies on single-center qualitative comparison.

#### 3.1.3 Cross-device image standardization

Differences in ultrasound equipment brands, probe frequencies, gain settings and scanning parameters will lead to significant differences in the grayscale, texture and visual performance of the same nodule (76). AI-based image standardization pipelines can unify image intensity distribution and spatial resolution, reducing systematic deviations caused by equipment differences. Such preprocessing provides a consistent data basis for subsequent multi-center model migration and clinical application. However, existing standardization algorithms are mostly validated on limited equipment types, and their stability in mixed-device clinical environments requires further verification.

### 3.2 Thyroid nodule segmentation and localization

Accurate nodule localization and pixel-level segmentation are essential prerequisites for automated nodule measurement, longitudinal growth monitoring and quantitative feature extraction (77). Traditional manual delineation is time-consuming and highly dependent on operator experience, resulting in inevitable inter-observer variability. AI automated segmentation and localization tools effectively improve the objectivity and efficiency of lesion identification in clinical workflows.

#### 3.2.1 Automatic nodule segmentation

U-Net and its improved variants are the most mainstream segmentation architectures for thyroid ultrasound lesions (78). With encoder–decoder structures and skip connection mechanisms, these models effectively capture multi-scale marginal details and achieve accurate segmentation of nodules with different sizes and morphologies. Most well-trained models obtain favorable DSC values under single-center test conditions, especially for nodules with relatively clear boundaries. Automated segmentation provides repeatable lesion boundary definitions, enabling objective volumetric measurement and longitudinal comparison of nodule changes during follow-up. Nevertheless, segmentation accuracy declines significantly for low-quality images, borderline lesions and nodules obscured by inflammatory parenchyma. At present, most segmentation models lack large-scale multi-center external validation, and their robustness in complex real-world imaging scenarios remains limited.

#### 3.2.2 Nodule detection and localization

Real-time detection frameworks such as YOLO and Faster R-CNN can automatically identify and frame focal lesions in ultrasound images, including tiny and easily overlooked micronodules (79, 80). Automated lesion localization assists junior sonographers in completing comprehensive scanning and reduces missed diagnosis rates in high-volume screening scenarios. This function is particularly valuable for population screening and multinodular goiter evaluation. However, most detection models are trained on high-quality tertiary hospital datasets, and their adaptability to low-end equipment and primary-care scanning environments has not been fully verified.

### 3.3 Automatic feature extraction and quantification

The subjective visual evaluation of TIRADS ultrasonic features inevitably causes inter-reader inconsistency, which affects the stability of clinical risk stratification (81). AI can quantitatively extract and objectively evaluate core ultrasonic markers, realizing standardized digital assessment of nodule sonographic characteristics.

#### 3.3.1 Core TIRADS marker quantification

Deep learning models can automatically identify and quantify multiple core malignant risk features, including calcification type, echogenicity, margin irregularity, aspect ratio and intranodular vascularity (82). Automated microcalcification detection achieves high sensitivity and specificity in retrospective cohort studies, showing stable auxiliary diagnostic capability (83). Different from subjective visual judgment, AI quantification evaluates pixel intensity distribution and edge morphological changes in a data-driven manner, effectively reducing human evaluation errors (84–86). Automated vascular signal analysis also provides quantitative evidence for auxiliary risk judgment (87). Even so, unified quantitative grading thresholds for these features have not been formed across different research institutions, and feature quantification standards remain heterogeneous.

#### 3.3.2 High-dimensional quantitative texture signatures

In addition to conventional TIRADS visual markers, AI and radiomic methods can extract high-dimensional texture features such as entropy, gray-level co-occurrence features and fractal features, which reflect microscopic tissue heterogeneity invisible to naked-eye observation (88). These subtle microstructural indicators provide supplementary diagnostic information for indeterminate Bethesda III–IV nodules with ambiguous conventional ultrasound manifestations, improving the differential diagnosis ability for gray-zone lesions (89). However, such high-dimensional features are highly dependent on accurate ROI segmentation and are prone to overfitting in small single-center datasets, making it hard to replicate stable diagnostic performance across diverse patient cohorts.

### 3.4 Benign-malignant differentiation

**Figure 4:**
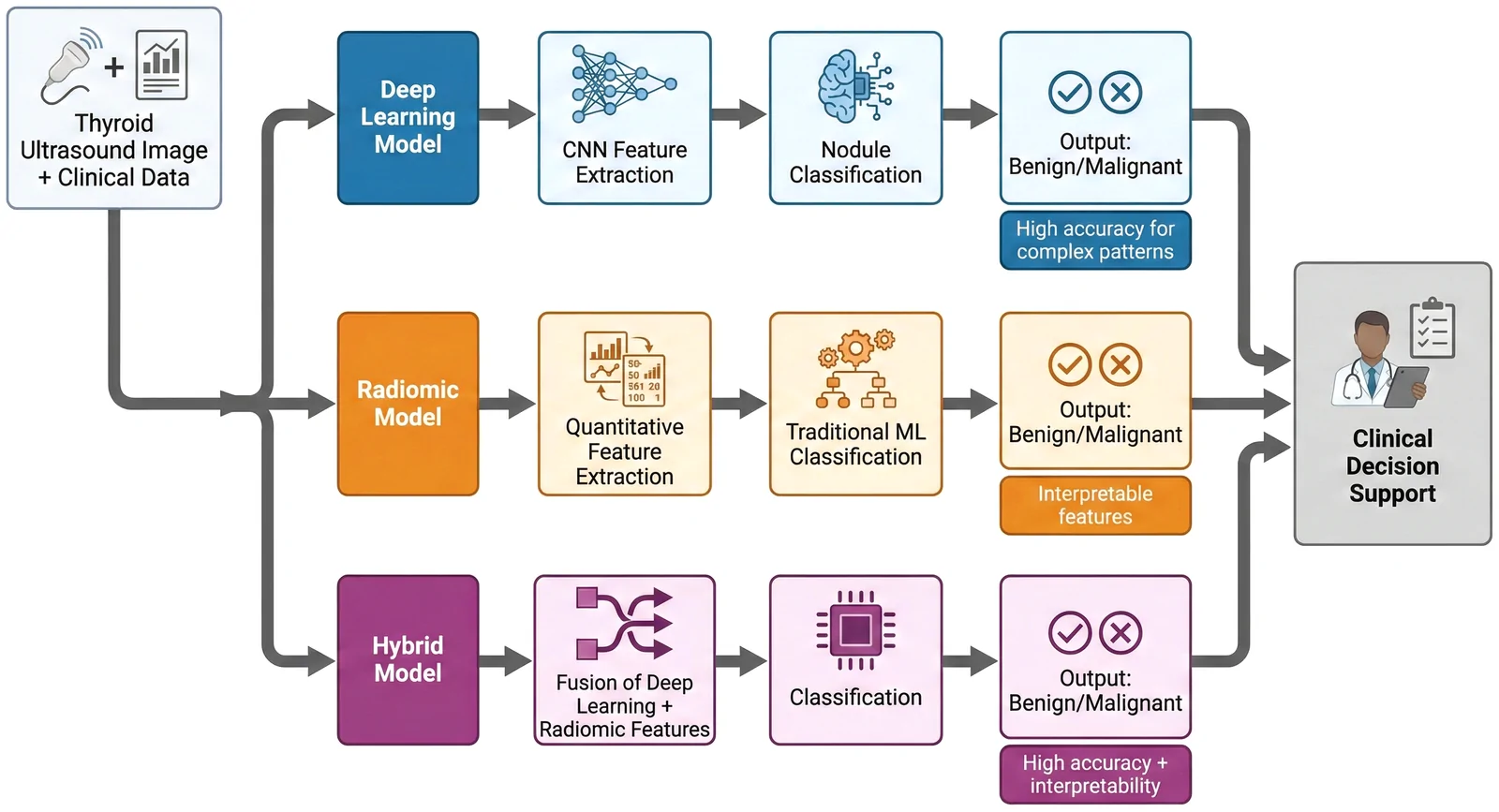
Three mainstream AI analytical pipelines for thyroid nodule benign-malignant differentiation.

The primary goal of thyroid ultrasound is to distinguish benign from malignant nodules, and AI models have shown great promise in this critical task (90). By integrating multi-dimensional features—including standard TIRADS features, quantitative radiomic features, and clinical variables—AI models can achieve diagnostic accuracy comparable to that of experienced radiologists, with the potential to reduce misdiagnosis and improve patient outcomes (**Figure 4**).

#### 3.4.1 Deep learning classification models

End-to-end CNN models directly learn hierarchical imaging patterns from raw ultrasound images to realize benign and malignant classification (91). Existing single-center and partial multi-center retrospective studies have shown that deep learning models achieve favorable AUC levels in nodule risk stratification, with diagnostic performance comparable to experienced sonographers under controlled experimental conditions (92). AI models show unique advantages in identifying microcarcinomas, lesions combined with Hashimoto’s thyroiditis, and cytologically indeterminate nodules (93). However, most models exhibit obvious performance degradation in external multi-center verification, due to inherent single-center selection bias and dataset heterogeneity.

#### 3.4.2 Radiomic classification models

Radiomic models rely on interpretable artificial features for risk prediction, possessing better result interpretability compared with black-box deep learning models (94). Radiomic signatures show good auxiliary value for indeterminate nodules and can effectively reduce unnecessary clinical biopsy rates. Nevertheless, most radiomic studies are limited to single-center retrospective designs, lacking prospective verification and multi-center external validation, and feature screening and modeling pipelines vary significantly among different studies.

#### 3.4.3 Hybrid deep learning-radiomics models

Hybrid models integrate deep learning high-level abstract features and radiomic interpretable features, combining the high discrimination performance of deep learning and the clinical interpretability of radiomics (95). Relevant studies have confirmed that hybrid models achieve better diagnostic performance than single models in borderline and micro-nodule cohorts. However, unified construction standards and reporting specifications for hybrid models are still lacking in this field, limiting standardized horizontal comparison and clinical promotion.

### 3.5 TIRADS classification optimization

**Figure 5:**
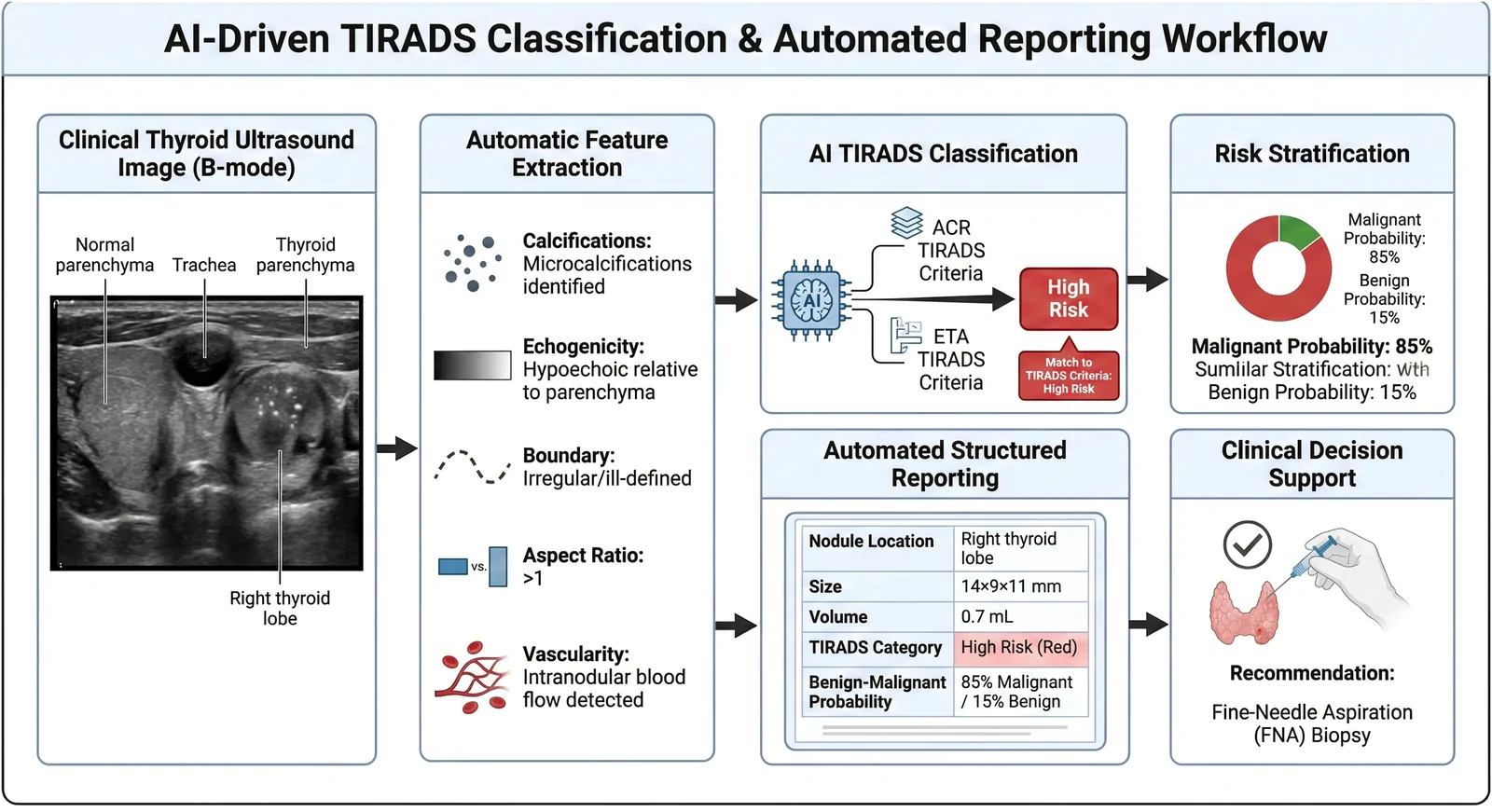
Complete AI-assisted TIRADS risk stratification and automatic reporting workflow.

A series of standardized thyroid nodule risk classification systems have been proposed worldwide, covering ACR-TIRADS, EU-TIRADS, K-TIRADS and domestic C-TIRADS (96). Each classification framework formulates independent feature scoring rules, risk stratification thresholds and corresponding clinical follow-up or biopsy recommendations based on regional epidemiological characteristics and clinical practice habits. When clinicians manually score nodules according to different TIRADS criteria, subjective weighting of ultrasonic features will lead to inconsistent risk stratification results for identical lesions, which brings confusion to clinical decision-making and long-term patient follow-up management. AI-assisted automatic grading pipelines solve this long-standing standardization problem by uniformly quantifying all ultrasonic risk markers and matching digital indicators with official scoring criteria of each TIRADS system (**Figure 5**).

Existing research verifies that TIRADS risk tiers output by AI models maintain high overall consistency with consensus diagnosis results jointly judged by multiple senior thyroid radiologists (97). The objective quantitative calculation mode eliminates subjective deviation in manual scoring, and the unified feature evaluation standard can unify diagnostic conclusions among different ultrasound practitioners and medical institutions. At present, most relevant model validation experiments take ACR-TIRADS as the main research object, while the research data supporting EU-TIRADS and C-TIRADS automatic grading is relatively insufficient, which restricts the cross-regional generalized application of such AI grading tools (96).

In addition to single ultrasonic image information, AI risk prediction systems can further incorporate multiple auxiliary predictive variables including patient age, serum thyroid function indicators and gene mutation markers to refine risk stratification of intermediate-risk TIRADS nodules (98). Such multi-dimensional combined models can effectively reduce the misclassification rate of grey-zone lesions between low-risk and high-risk tiers. However, most optimized multi-factor models are trained based on geographically limited single-center patient cohorts, without large-scale cross-population external validation, and their predictive stability for patient groups with different demographic and pathological characteristics cannot be guaranteed. Besides, most published literatures fail to systematically elaborate the detailed matching logic between AI quantified ultrasonic features and official TIRADS scoring rubrics, which reduces the repeatability of related research results and hinders the standardized optimization of subsequent grading algorithms.

### 3.6 Automated reporting and clinical decision support

Manual compilation of structured ultrasound diagnostic reports occupies massive clinical labor in high-volume ultrasound departments. AI systems can automatically generate standardized diagnostic documents and output evidence-based clinical management suggestions based on quantified nodule malignant risk data (99).

#### 3.6.1 Structured automated reporting

AI pipelines integrate all measurable clinical information including nodule location, three-dimensional diameter, volume, quantified ultrasonic risk markers, TIRADS risk grade and predicted malignant probability to generate complete diagnostic reports that comply with clinical guideline requirements (100). Automated reporting shortens documentation time and avoids omission of key diagnostic indicators, and standardized report content facilitates longitudinal data comparison within electronic medical record systems. The primary barrier to large-scale clinical deployment lies in the lack of unified report templates across different medical institutions and imaging platforms.

#### 3.6.2 Clinical decision support

Based on comprehensive nodule risk stratification results, embedded AI auxiliary modules output standardized clinical suggestions covering FNA biopsy indications, follow-up surveillance intervals and surgical referral criteria (101). Such auxiliary functions provide reliable reference for clinicians without professional thyroid imaging training, especially in resource-limited primary medical institutions. It must be emphasized repeatedly that all algorithmic suggestions only serve as auxiliary reference, and the final diagnosis and treatment decisions need to be confirmed by licensed professional clinicians. Most existing clinical decision support prototypes are only tested in tertiary referral hospitals, and their practical clinical utility and potential bias under primary care workflow have not been systematically assessed.

## 4 Limitations and clinical challenges of AI technology

Promising auxiliary performance of thyroid ultrasound AI tools is mostly demonstrated under single-center retrospective research conditions, while multiple inherent technical bottlenecks, clinical translation obstacles, and unresolved ethical & regulatory constraints still restrict stable large-scale deployment in real-world routine clinical practice (102). As a narrative review without quantitative bias assessment or systematic evidence pooling, this chapter qualitatively summarizes universal drawbacks observed in published thyroid AI research and analyzes the root causes of these translational barriers based on existing literature.

### 4.1 Inherent technical limitations of current AI frameworks

Most thyroid ultrasound AI prototypes are restrained by interconnected dataset limitations, opaque deep learning architectures and weak adaptability to low-quality clinical scans, alongside frequent overfitting issues in small-scale single-center training cohorts (103).

The majority of thyroid AI training and validation datasets are sourced from single tertiary hospitals, which inevitably introduce selection bias shaped by regional patient demographics, limited ultrasound equipment types and localized nodule spectrum (104). Models that achieve satisfactory diagnostic metrics on internal test sets often show obvious performance degradation when validated on external multi-center cohorts, a phenomenon known as dataset shift (105). Multi-center collaborative data collection can partially relieve this issue, yet globally shared standardized annotated thyroid ultrasound databases remain scarce, and unified multi-institutional labeling incurs huge time and labor costs.

Nearly all datasets face severe pathological category imbalance. Follicular carcinoma, medullary carcinoma, anaplastic carcinoma and atypical inflammatory benign nodules occur at low clinical incidence, so their sample proportions are extremely low within training cohorts (106). Insufficient representative samples make it hard for models to capture unique ultrasonic signatures of rare lesions, increasing risks of misdiagnosis and missed diagnosis in routine scanning. Balanced sampling for rare pathological subtypes has not become a standard modeling step in most published studies.

End-to-end CNN deep learning models are typical black-box architectures without traceable feature reasoning logic (107). Clinicians cannot identify which ultrasonic markers drive malignant risk prediction, reducing trust in AI outputs and creating ambiguous liability risks once diagnostic errors occur. Radiomics improves interpretability via handcrafted quantitative features, but there is an unavoidable trade-off between classification accuracy and result transparency across current research.

The stability of all AI algorithms heavily relies on standardized high-quality ultrasound images acquired under strict scanning specifications in tertiary hospitals (108). Motion blur, acoustic shadow and uneven gain widely seen in grassroots and emergency scans will severely interfere with segmentation, feature extraction and benign-malignant discrimination. No mature adaptive correction algorithm can fully offset the negative impact of substandard images, and most research only carries out verification on high-quality standardized pictures without real-scene mixed-image testing.

Overfitting is another common problem for models trained on limited single-center imaging data (109). Neural networks tend to memorize noise and unique artifact features in training images instead of universal pathological imaging patterns. Even with regularization and data augmentation strategies, overfitting cannot be completely avoided. Such models often obtain excellent indicators in internal cross-validation but behave unstably on unseen independent test cohorts, greatly weakening their value for cross-hospital clinical application.

### 4.2 Practical barriers hindering clinical translation

Laboratory AI prototypes encounter multiple practical obstacles when migrated to routine hospital workflows, including information system incompatibility, strict regulatory thresholds, uneven applicability across tiered medical institutions and insufficient clinician training support.

Most AI algorithms developed in laboratories exist as independent offline programs and cannot be seamlessly embedded into mainstream ultrasound equipment, PACS and electronic medical record systems (110). Sonographers need to export images separately and manually copy AI analysis results into diagnostic reports, adding redundant operations instead of lowering clinical burden. Globally unified data interface standards for docking AI and imaging hardware have not been established, leading to high information reconstruction costs for hospitals that intend to deploy such tools.

The research transformation of thyroid ultrasound AI needs to comply with strict supervision rules issued by authorities such as FDA and EMA (111). Large-scale prospective multi-center clinical trials are mandatory for market approval, which consume massive financial and time resources. Besides, the iterative updating characteristic of AI algorithms conflicts with static supervision standards for traditional medical devices, and unified post-market re-verification specifications for updated models are absent across different regions.

The practical auxiliary value of AI varies significantly according to clinicians’ experience levels. For senior thyroid radiologists, AI only brings limited improvement in diagnostic efficiency; for junior physicians and grassroots practitioners lacking systematic training, automated lesion detection effectively reduces missed diagnosis of tiny nodules. However, nearly all model validation cohorts are sourced from tertiary hospitals, lacking sufficient real-world data to assess their effects and potential bias in primary care screening. Meanwhile, the construction and long-term maintenance of AI systems require continuous capital investment, forming deployment barriers for resource-limited medical centers (112).

Many clinical practitioners hold reserved attitudes toward AI auxiliary diagnosis (113). Some senior clinicians worry that over-reliance on algorithms will weaken their independent clinical judgment, so they are reluctant to apply AI tools in routine diagnosis. Even clinicians willing to try AI lack standardized training covering model applicable scope, limitations and result interpretation methods (114). At present, systematic targeted training courses for thyroid AI are not popularized, and few studies evaluate clinicians’ real acceptance under routine clinical workflows.

### 4.3 Ethical, legal and fairness-related hidden risks

Large-scale collection of patient thyroid imaging data and popularization of AI diagnosis bring unresolved privacy protection, liability division and patient informed consent issues.

Model training and iterative optimization rely on massive thyroid ultrasound images containing sensitive personal medical information (115). In multi-center data sharing and collaborative research, incomplete de-identification may lead to patient privacy leakage. Unified mandatory industry specifications for the storage, transmission and authorized secondary use of imaging research data have not been formulated. Many published papers fail to fully report ethical approval documents and complete patient informed consent records, bringing potential compliance risks to relevant clinical studies.

Existing domestic and international medical legal systems have not issued clear normative provisions to define accountability when AI outputs induce missed or delayed thyroid cancer diagnosis (116). If clinicians fully defer to AI high-risk prompts and ignore other suspicious ultrasonic signs, it is difficult to allocate responsibility among attending physicians, algorithm developers and medical equipment suppliers. Vague liability boundaries make most hospitals adopt a wait-and-see attitude toward large-scale AI deployment.

Training datasets with insufficient population diversity will cause systematic diagnostic bias against children, pregnant women and the elderly (117). Such imbalance will further widen existing medical disparities, yet fairness evaluation is rarely set as a necessary verification link in current AI research design.

Most patients cannot receive standardized clear notification that AI will participate in their thyroid image analysis (118). Information asymmetry between doctors and patients hinders comprehensive public understanding of AI’s auxiliary positioning and inherent defects. Non-standard informed consent documents for reusing patient imaging data also reduce patient trust in AI diagnostic services.

## 5 Future perspectives

The limitations summarized above restrict the large-scale clinical promotion of thyroid ultrasound AI tools. To address these bottlenecks and further expand the clinical value of relevant technologies, researchers have put forward multiple promising development directions based on existing research evidence (119). This narrative review qualitatively sorts out feasible technical optimization paths, expanded clinical application scenarios and unified standardization construction ideas, without quantitative prediction or pooled analysis of relevant research data.

### 5.1 Technical development directions

A series of targeted technical innovations are being explored to resolve data bias, poor interpretability and insufficient real-time performance of existing AI models.

Multi-modal data fusion is one core development trend. Traditional thyroid ultrasound AI only relies on single B-mode grayscale images for analysis, while multi-modal fusion schemes integrate elastography, nuclear thyroid imaging, CT/MRI, serum thyroid function indicators, gene mutation markers and genomic data with ultrasound features (120). Supplementary multi-dimensional information can effectively improve the differential diagnosis efficiency of indeterminate Bethesda III–IV nodules and rare malignant subtypes. At present, most fusion-related research remains single-center exploratory work, lacking unified fusion frameworks and large-scale multi-center prospective verification.

Explainable artificial intelligence (XAI) is expected to break the black-box limitation of deep learning (121, 122). Common XAI technologies include feature heatmap visualization, attention weight analysis and natural language interpretation modules. These tools can mark the ultrasonic lesions and features that dominate the model’s risk judgment, helping clinicians understand the reasoning logic of AI outputs, improving clinical trust and meeting the transparency requirements of medical device regulatory review. However, unified quantitative evaluation indicators for XAI effects have not been formed in the industry, and relevant reporting specifications are not uniform among different research teams.

Real-time point-of-care AI systems represent another important research direction (123). Such algorithms can be embedded directly into front-end ultrasound machines, providing synchronous nodule detection, feature quantification and risk prompts during scanning, without post-image export and offline analysis. Real-time auxiliary functions can effectively assist novice operators to complete standardized image acquisition, which is especially suitable for grassroots large-scale population screening. But most prototype real-time AI systems are only verified in tertiary hospitals, lacking stability tests under limited-resource clinical environments.

Federated learning and privacy-preserving learning technologies can simultaneously solve data scarcity and patient privacy protection difficulties (124). This training mode allows multiple medical institutions to jointly iterate AI models without transmitting raw patient ultrasound data, avoiding the risk of privacy leakage caused by cross-hospital data sharing and expanding the diversity of training datasets to reduce dataset shift. Small-sample and few-shot learning algorithms further lower the annotation threshold for rare nodule subtypes, yet the practical multi-center collaborative application cases of such technologies are still scarce, and unified implementation standards have not been established.

**Figure 6:**
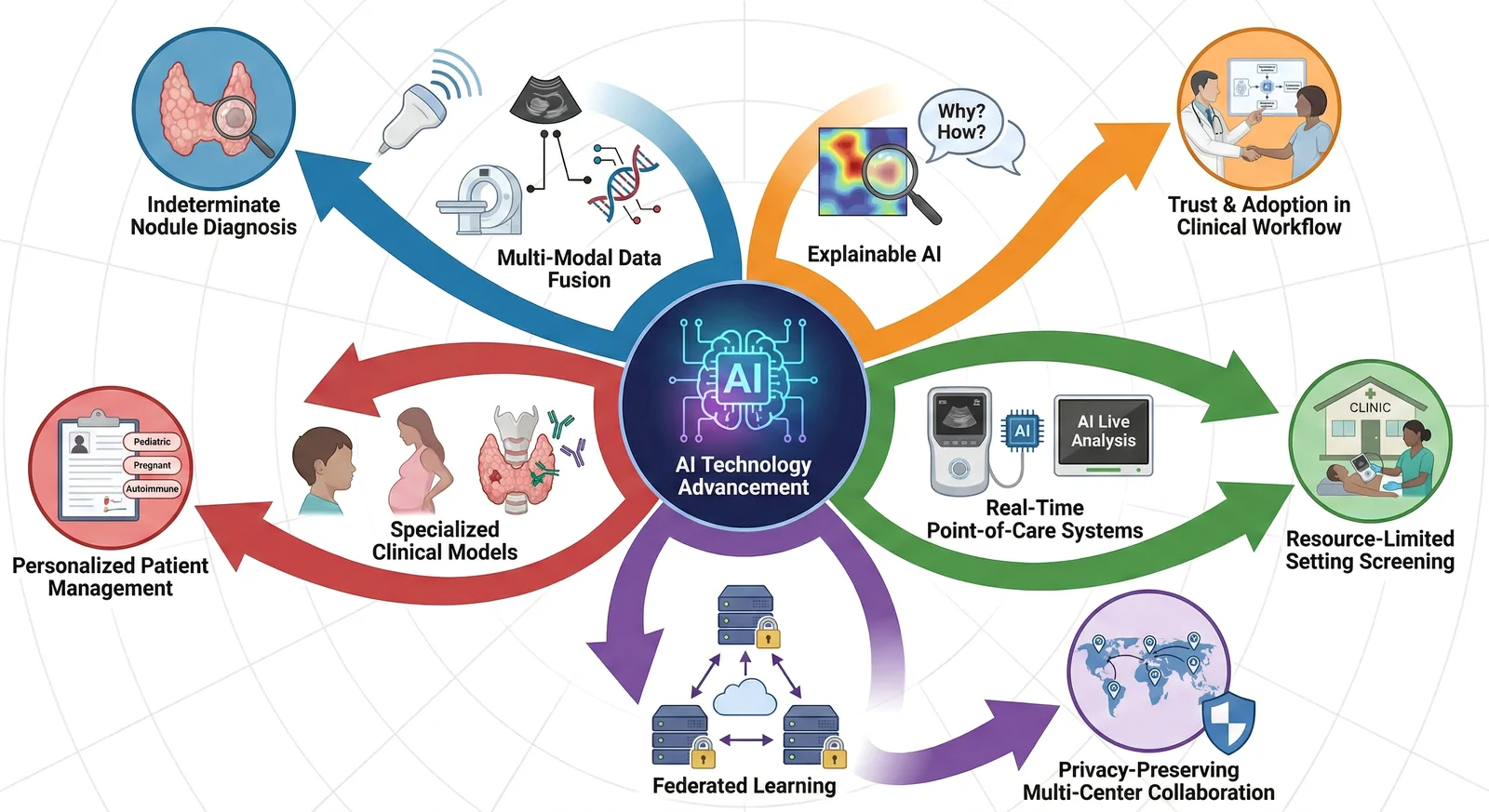
Schematic overview of core AI technical innovations and matching clinical application scenarios.

The above five major technical evolution directions are systematically summarized in (**Figure 6**), which visually demonstrates the logical connection between advanced AI technologies and diversified clinical application demands.

### 5.2 Expansion of targeted clinical application scenarios

Future AI research will no longer be limited to routine benign and malignant differentiation of general thyroid nodules, and more specialized models for specific patient groups and full-cycle disease management will be developed.

Customized AI models for special populations including children, pregnant women and patients with chronic autoimmune thyroiditis are urgently needed (125, 126). These groups have unique thyroid anatomical characteristics, lesion distribution rules and disease risk characteristics different from ordinary adults; general-purpose models often show reduced diagnostic accuracy when applied to them. Current targeted research mostly relies on single regional patient cohorts, and cross-population external verification data is insufficient.

Predictive and prognostic AI systems will gradually mature (127). Different from static nodule risk classification, such models combine serial follow-up ultrasound images, molecular markers and clinical indicators to predict the long-term progression risk of nodules, tumor invasion possibility and postoperative recurrence probability, providing personalized active surveillance or surgical treatment suggestions for patients. Existing prognostic research is mostly based on retrospective single-center data, lacking long-term prospective follow-up evidence support.

Deep integration of AI and telemedicine will improve the accessibility of thyroid diagnosis in remote and under-resourced areas (128). Portable ultrasound equipment matched with lightweight AI algorithms can transmit imaging data and real-time auxiliary analysis results to central expert platforms for remote consultation, alleviating the shortage of professional thyroid ultrasound physicians in grassroots medical institutions. At present, the field lacks unified operation specifications and large-scale field application data of telemedicine-AI combined systems.

AI will evolve into a complete supporting tool for full-cycle thyroid disease management, covering initial screening, risk stratification, intervention guidance and long-term follow-up monitoring, rather than only serving single imaging diagnosis (129). Integrated disease management platforms based on ultrasound AI can automatically sort follow-up plans according to nodule risk grades, realize intelligent reminder of patients, and summarize longitudinal lesion change trends. Related integrated management systems are still in the initial research stage, with limited real clinical deployment experience.

### 5.3 Standardization and regulatory system construction

Unified industry standards and complete regulatory norms are the necessary prerequisites for safe, standardized and generalized clinical application of thyroid ultrasound AI technology.

The construction of publicly shared standardized multi-center annotated datasets and unified image annotation guidelines is the primary foundation (130, 131). Consistent marking rules for nodule boundaries, calcification subtypes and pathological labels can eliminate data incompatibility between different research teams, lay a unified training foundation for cross-institutional generalizable models. At present, the global public high-quality thyroid ultrasound annotated resource bank is extremely scarce, and the annotation standards adopted by various research institutions are inconsistent.

Uniform model validation guidelines and comprehensive performance evaluation metrics need to be formulated (132). Clear minimum sample size requirements for internal and external verification, mandatory multi-center prospective trial thresholds and standardized diagnostic evaluation indicators should be clarified to realize horizontal fair comparison of different AI models. The current evaluation systems of various literatures differ greatly, which interferes with the objective synthesis of research evidence.

Regulatory frameworks for medical AI devices need to be adjusted to adapt to the iterative updating characteristics of algorithms (133). New supervision modes targeting continuous model optimization after listing should be formulated, including regular real-world data re-verification mechanisms. At present, the approval standards for thyroid ultrasound AI products are inconsistent in different regions of the world, bringing obstacles to cross-regional clinical promotion.

Professional medical societies need to release unified clinical application guidelines for AI auxiliary diagnosis (134). The guidelines should clarify the applicable scope, limitations and standardized operation flow of AI tools, formulate systematic training plans for ultrasound clinicians, and standardize the way AI results are recorded in diagnostic reports. Up to now, unified industry-wide AI application consensus has not been fully popularized in endocrinology and radiology.

Long-term clinical quality control and assurance mechanisms should be established in medical institutions (135). Hospitals deploying AI systems need to regularly monitor model performance drift caused by changes in patient population and equipment, carry out periodic comparative verification with expert diagnosis results, and form standardized adverse feedback channels for algorithm errors. At present, most medical institutions have not built targeted AI long-term quality control systems.

## 6 Conclusions

Throughout this narrative review, we systematically summarize the full-range auxiliary functions of artificial intelligence in thyroid ultrasound workflows, while objectively analyzing widespread methodological defects and translational obstacles observed in existing published research (136). Relevant AI tools deliver apparent advantages under single-center retrospective research conditions, yet multiple technical, clinical and regulatory hurdles still block their stable, large-scale deployment in routine clinical settings.

When tested under single-center retrospective research conditions, AI delivers tangible auxiliary improvements covering the full thyroid ultrasound diagnostic workflow. From raw ultrasound image optimization and automated lesion segmentation, to quantitative feature measurement, standardized TIRADS risk stratification and intelligent structured reporting, AI effectively mitigates long-standing clinical pain points including subjective inter-observer discrepancy and heavy manual documentation workload (137). For subtle microcarcinomas and indeterminate Bethesda III–IV nodules with ambiguous sonographic manifestations, AI-assisted analysis provides extra objective imaging evidence to reduce unnecessary invasive examinations and repeated biopsies, which can optimize overall medical resource allocation, especially in resource-limited primary care facilities short of experienced thyroid radiologists.

Nevertheless, the majority of available supporting evidence is derived from single-center retrospective cohorts. Most current AI prototypes lack large multi-center prospective external validation, and inconsistent experimental pipelines lead to poor cross-study comparability, which greatly weakens the reliability of existing research evidence. Core technical drawbacks remain unresolved, including severe dataset selection bias, insufficient samples of rare pathological subtypes, poor model interpretability and heavy reliance on high-quality standardized ultrasound images. Meanwhile, disconnection between AI algorithms and hospital information systems, ambiguous medical liability standards for algorithm-induced misdiagnosis, and imperfect data privacy supervision jointly form multi-dimensional barriers restricting real-world clinical translation.

Looking ahead, the sustainable development of thyroid ultrasound AI relies on coordinated breakthroughs in multiple dimensions. Future research should prioritize multi-modal data fusion, explainable AI frameworks, privacy-preserving federated learning and lightweight real-time scanning systems to tackle inherent model defects. In parallel, unified global annotation criteria, standardized multi-center validation protocols and complete medical AI regulatory systems need to be established to narrow the gap between laboratory algorithms and daily clinical practice. Specialized models tailored to children, pregnant women and patients with autoimmune thyroiditis, as well as predictive prognostic tools for tumor progression, will further expand the application boundary of AI beyond simple nodule benign-malignant discrimination, realizing full-cycle intelligent thyroid disease management.

It is critical to repeatedly clarify that artificial intelligence only serves as an auxiliary reference tool rather than a complete replacement for professional clinicians. Final diagnosis, risk stratification and personalized treatment decisions must rely on comprehensive clinical judgment integrating imaging findings, laboratory indicators, patient medical history and pathological results. The complementary collaboration between clinicians and AI systems can minimize human diagnostic bias and improve the accuracy and consistency of thyroid nodule assessment. Subsequent relevant studies should strictly adopt prospective multi-center research designs and follow unified AI reporting specifications to generate higher-level clinical evidence and promote standardized, equitable AI application across all tiers of medical institutions.
